# An experimental study on the mediating roles of career decision-making self-efficacy between digital media literacy and employment choices of university students

**DOI:** 10.3389/fpsyg.2026.1963363

**Published:** 2026-10-22

**Authors:** Shuang Wu

**Affiliations:** School of Finance, Dongbei University of Finance and Economics, Dalian, Liaoning, China

**Keywords:** algorithmic awareness for careers, bidirectional mediation, career decision making self efficacy, digital media literacy, factorial randomized controlled trial

## Abstract

Algorithmic recommendation now shapes the career information university students see, yet whether raising students' understanding of these algorithms improves employment choices remains unclear. This study tested a dynamic bidirectional mediation model linking digital media literacy, career decision making self efficacy (CDMSE), and employment choices, with Algorithmic Awareness for Careers (AAC) specified as a distinct subdimension of digital media literacy. In a 2 × 2 factorial randomized controlled trial, 204 graduating undergraduates from three universities in southern China were assigned to Media Critical Literacy Training (MCLT), Career Algorithm Transparency Training (CATT), both, or neither; 192 completed four assessment waves ending 6 months after graduation. CATT mainly raised AAC (partial η^2^ = 0.128), MCLT mainly raised critical literacy (partial η^2^ = 0.096), and the two components jointly strengthened CDMSE. Parallel process latent growth modeling showed that AAC growth predicted CDMSE growth (β = 0.42), which in turn predicted later AAC [β = 0.28, 95% CI (0.13, 0.43)], and that CDMSE carried a significant indirect effect to a composite employment outcome [0.151, 95% CI (0.077, 0.238)]. Relative to the control condition, the combined condition was associated with better outcomes 6 months after graduation, including more document verified offers, an 18.1% higher starting salary, closer externally coded major job match, and greater employment satisfaction. The findings identify AAC as a trainable component of digital media literacy, empirically operationalize the bidirectional feedback assumed by social cognitive career theory, and support embedding algorithm transparency training in university career education.

## 1 Introduction

The number of university graduates in China was projected to reach a record 12.22 million in 2025, 430,000 more than in the previous year (Ministry of Education of the People's Republic of China, 2024), and the surveyed unemployment rate among urban youth aged 16 to 24 reached 21.3% in June 2023, although the bureau later excluded enrolled students from this series (National Bureau of Statistics of China, 2023, 2024). Under this pressure, short video platforms, algorithmic content feeds, and professional social networking platforms are replacing traditional recruitment presentations as the main channels through which students examine industries, employers, and positions (Chen et al., 2025).

Because algorithms decide which content reaches students and in what amount, digital media literacy has become a critical form of individual capital in career choice, and it is positively associated with employment related outcomes, with career self efficacy as a key transmission mechanism (Wang et al., 2024b). According to social cognitive career theory (SCCT), career information encountered during exploration is a learning experience that shapes career decision making self efficacy (CDMSE), and CDMSE in turn guides goals and actions (Lent et al., 2022; Wang et al., 2022). Digital media literacy, especially the ability to recognize and manage algorithmic filtering, may therefore influence employment choices through the efficacy beliefs it helps to build.

Although research has begun to connect these constructs, three gaps prevent a clear answer. Existing measures of algorithmic literacy address general internet and social media use rather than career information (Dogruel et al., 2022; Oeldorf-Hirsch and Neubaum, 2025; Shin et al., 2022), and research on algorithmic hiring has focused on employers rather than on how job seekers understand the filtering of career information (Hunkenschroer and Luetge, 2022; Langer and König, 2023). Evidence that CDMSE transmits the effects of personal resources to career outcomes comes largely from cross sectional surveys that treat efficacy as a one way conduit (Xiong et al., 2025; Zhou et al., 2024), so the feedback from efficacy to the information environment assumed by SCCT has rarely been tested. Intervention studies have also seldom followed their effects beyond the posttest (Wang et al., 2024a; Zheng et al., 2025). It therefore remains unclear whether training students to understand algorithmic filtering can strengthen CDMSE and, through it, improve the employment outcomes that graduates actually obtain. Such evidence is needed now that algorithm literacy is being proposed as part of digital competence frameworks (Moylan and Code, 2024).

To address these gaps, the present study separates Algorithmic Awareness for Careers (AAC) from digital media literacy, operationalizes it as students' metacognitive capacity to understand how recommendation algorithms filter career information, and examines it in a 2 × 2 factorial randomized trial with four measurement waves ending six months after graduation. The ultimate aim of the study is to test a dynamic bidirectional mediation model in which digital media literacy, and AAC in particular, improves employment choices through CDMSE, while growth in CDMSE in turn strengthens AAC.

## 2 Literature review

### 2.1 Digital media literacy and algorithmic awareness for careers

Digital media literacy refers to the capacities to access, evaluate, and create media content. Mainstream scales follow a three part framework of information literacy, critical reading, and content creation, so although algorithmic systems act as gatekeepers of career information, algorithmic awareness has rarely been measured as a separate subdimension, even though multidimensional AI literacy scales treat algorithmic influence as a core component (Yuan et al., 2024). Algorithmic awareness shapes the breadth of users' information use (Petrovčič et al., 2025) and interacts with their own information protection strategies (Erickson, 2024), so a framework of tool use plus information discernment cannot fully explain which career opportunities students see and how they come to see them.

Existing constructs of algorithmic awareness and algorithm literacy are the conceptual starting point for AAC, but AAC differs from them in domain, object, and function. General constructs concern the curation of news, entertainment, and advertising and whether users know that algorithms operate and use personal data (Dogruel et al., 2022; Gruber and Hargittai, 2023; Voorveld et al., 2024), and they have mainly been studied as correlates of attitudes, privacy concerns, and persuasion coping (Oeldorf-Hirsch and Neubaum, 2023; Shin et al., 2022). AAC instead concerns job postings, employer content, and occupational information, a domain in which algorithms increasingly mediate recruitment (Hunkenschroer and Luetge, 2022). It captures whether students recognize that algorithmic filtering shapes, and may leave incomplete, the career options visible to them, and it serves as a self regulatory resource that includes diversifying career information and critically evaluating algorithmic job recommendations. Unless AAC is separated as a distinct subdimension alongside information, critical, and creation literacy, career choice models that treat digital media literacy as a predictor omit a crucial segment of the causal chain.

### 2.2 Career decision making self efficacy

CDMSE is the confidence that one can complete the tasks required for a career decision, including appraising oneself, gathering occupational information, selecting goals, planning, and solving problems (Betz et al., 1996). SCCT holds that self efficacy beliefs develop from learning experiences and guide goals and actions, while performance attainments become new learning experiences that feed back into efficacy (Wang et al., 2022), and in the career self management model, process efficacy predicts goals and exploratory actions (Lent et al., 2022). Mediation models, many based on Chinese university students, have repeatedly verified the transmission effect of CDMSE (Xiong et al., 2025; Zhou et al., 2024), including over up to 20 months in a longitudinal study (Kvasková et al., 2023).

Building on Bandura's triadic reciprocal framework, SCCT emphasizes bidirectional feedback among person, behavior, and environment, yet reviews note that existing models rarely operationalize the spiral through which the environment reshapes efficacy and efficacy in turn filters the environment (Wang et al., 2022; Yao and McWha-Hermann, 2025). Most mediation tests use cross sectional data and treat CDMSE as a one way conduit (Agoes Salim et al., 2023; Cheung, 2024; Karacan Ozdemir and Buyukcolpan, 2024), so the mediator needs to be modeled as a dynamic bidirectional process with the feedback loop of efficacy at the center of the analysis.

### 2.3 Employment choices

Employment choices include the preferences that direct a job search, such as location, organizational type, industry, and match with one's major, and the outcomes that the search produces, such as job offers, starting salary, major job match, and employment satisfaction. Digital literacy has been linked to employment related outcomes: among Chinese university students, it relates to career competence (Chen et al., 2025), and digital information literacy self efficacy partially mediates the link between psychological adaptability and work engagement (Gao et al., 2025). Evaluations of career interventions, however, seldom reach the outcome side. Effects are usually evaluated with self reported intentions, CDMSE scores, or short term decision efficacy rather than with offers or employment after graduation, and few interventions include follow up assessments or examine long term outcomes (Wang et al., 2024a; Zheng et al., 2025). Whether stronger digital media literacy leads to better employment choices has therefore been examined mainly through intentions rather than through the jobs that graduates actually obtain.

### 2.4 Linking the three constructs and purpose of the study

The three constructs are linked through the triadic reciprocal logic of SCCT. In the triadic scheme marked in Figure 1, CDMSE represents the personal factor, employment choices the behavioral factor, and digital media literacy with its AAC component the way students engage the algorithmic information environment, that is, the environmental factor. Digital media literacy thus shapes the learning experiences from which CDMSE develops, CDMSE guides employment choices, and the same logic implies a return path, because efficacy changes how students attend to and select information.

**Figure 1:**
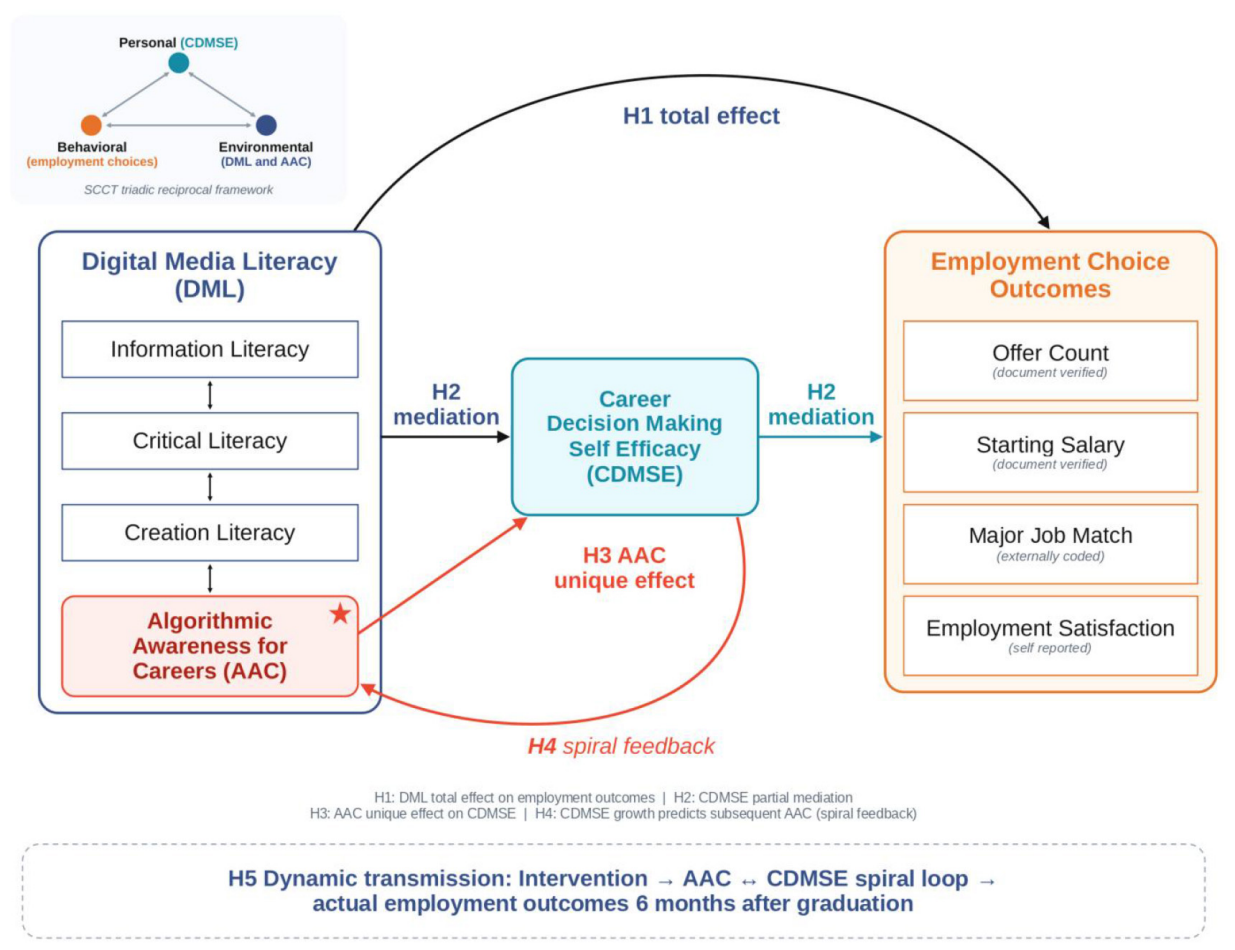
SCCT based dynamic bidirectional mediation framework and research hypotheses for digital media literacy, career decision making self efficacy, and employment choice.

Existing evidence cannot yet test this loop. Job search self efficacy research relies mostly on cross sectional designs with simple mediation and almost no causal chains (Zheng et al., 2025), which cannot identify the linking process when the order of variables is not secured by design (Rohrer et al., 2022). Multiwave studies establish the temporal order of naturally changing variables (Liu et al., 2024; Yi et al., 2025) but cannot show whether raising self efficacy through instruction would alter later outcomes, and the few intervention studies rarely include randomized controls or multiple follow up occasions (Wetstone and Rice, 2023). The purpose of this study is therefore to test, in a randomized factorial experiment with four measurement waves, whether AAC and CDMSE develop reciprocally and jointly transmit training effects to the employment outcomes that graduates obtain. Five hypotheses follow from this purpose: digital media literacy predicts the quality of employment choices (H1); CDMSE partially mediates this relation (H2); AAC contributes more to CDMSE than the other subdimensions do (H3); growth in CDMSE feeds back into later AAC (H4); and training effects reach actual employment outcomes through this reciprocal process (H5). The next section develops each hypothesis from the reviewed evidence.

## 3 Hypothesis development

The hypotheses translate this framework into testable relations. At its core, digital media literacy is reconceptualized as four subdimensions: information literacy, critical literacy, creation literacy, and AAC, a distinct subdimension that extends general algorithmic awareness to career information.

### 3.1 Digital media literacy and employment choices

Students who can locate, verify, and interpret career information are better placed to identify suitable vacancies, as the associations of digital literacy with career competence and work engagement suggest, and algorithmic curation makes these capacities more consequential because it determines which options become visible. Within SCCT, this prediction corresponds to the pathway from learning experiences to career outcomes.

H1: Digital media literacy positively predicts the quality of employment choices.

### 3.2 The mediating role of career decision making self efficacy

Digital media literacy, and AAC in particular, is a learning resource that determines which career information students encounter and how they interpret it, and CDMSE is the process efficacy belief that SCCT places between learning experiences and career actions. Digital media literacy may also act on employment choices directly, because a wider and better verified set of visible vacancies can improve outcomes regardless of efficacy, so CDMSE is expected to carry part, but not all, of the effect. Within SCCT, this hypothesis represents the efficacy mediated part of the model.

H2: Career decision making self efficacy partially mediates the relationship between digital media literacy and the quality of employment choices.

### 3.3 The domain specific contribution of AAC

Efficacy beliefs are domain specific. Awareness of how recommendation systems filter job information is the subdimension most proximal to career decisions and shapes how broadly users search for and use information (Gruber and Hargittai, 2023; Petrovčič et al., 2025), whereas information, critical, and creation literacy apply to many media tasks and should contribute less to CDMSE. Within SCCT, AAC thus represents the domain specific source of efficacy.

H3: The independent effect of AAC on CDMSE is significantly greater than the effects of the other subdimensions.

### 3.4 Reciprocal feedback from career decision making self efficacy to AAC

Triadic reciprocity also runs from efficacy to the environment, because people select and engage their environments according to what they believe they can do. Students who become more confident about career decisions are more likely to search actively, compare sources, and adjust recommendation settings, behaviors that accompany algorithmic awareness among social media users (Oeldorf-Hirsch and Neubaum, 2023). Because recommendation systems respond to such behavior, growth in efficacy should in turn raise awareness of algorithmic filtering.

H4: Growth in CDMSE positively predicts subsequent AAC, thereby forming spiral feedback.

### 3.5 Transmission to actual employment outcomes

If AAC and CDMSE reinforce each other, an intervention that raises AAC should influence employment outcomes mainly through this reciprocal growth rather than through isolated change in either construct, and its consequences should appear beyond intentions, in the jobs that graduates obtain.

H5: Intervention effects are transmitted through the dynamic spiral of AAC and CDMSE to actual employment outcomes at 6 months after graduation.

The framework and hypotheses are shown in Figure 1.

## 4 Method

### 4.1 Research design

The study used a 2 × 2 factorial randomized controlled design that crossed Media Critical Literacy Training (MCLT) with Career Algorithm Transparency Training (CATT), yielding four conditions. Decomposing the Media Integrated Career Planning Curriculum (MICPC) into these two orthogonally combinable components estimates their independent main effects and interaction simultaneously and shows which component is responsible for an effect rather than only whether the whole curriculum works (White et al., 2024).

MCLT trains the critical decoding of career related media content, including multisource verification of career information in short videos, credibility assessment of employment information, differentiation between promotional narratives and structured information, and self regulation of social comparison. CATT trains metacognitive understanding of algorithmic recommendation and active intervention, including visual transparency training, diversification of recommended information, evaluation of AI assisted career exploration tools, and identification of algorithmic discrimination and information cocoons. Each module comprised 8 instructional h, with MCLT delivered in the first 4 weeks and CATT in the last 4 weeks of the eight week intervention (Table 1).

**Table 1:** Intervention content and total duration of the four conditions in the 2 × 2 factorial design.

Condition code	Combination	MCLT module	CATT module	Total intervention duration
*A*_1_ *B*_1_	MCLT+CATT	Critical interpretation, multisource verification, credibility assessment, and regulation of social comparison (8 instructional h)	Algorithm visualization, recommendation diversification, AI tool evaluation, and information cocoon identification (8 instructional h)	16 instructional h
*A*_1_ *B*_0_	MCLT+Control	Same as above (8 instructional h)	General career planning lectures (8 instructional h)	16 instructional h
*A*_0_ *B*_1_	Control+CATT	General career planning lectures (8 instructional h)	Same as above (8 instructional h)	16 instructional h
*A*_0_ *B*_0_	Control+Control	General career planning lectures (8 instructional h)	General career planning lectures (8 instructional h)	16 instructional h

The control condition consisted of general career planning lectures matched for total exposure, so that every condition received 16 instructional h. Instructors, class schedules, and the visual design of teaching materials were held constant across conditions.

### 4.2 Participants and sample size

Participants were graduating undergraduates at three comprehensive universities in southern China. The sample size was estimated for a fixed effects ANCOVA with a small to medium effect size, α = 0.05, and power of 1–β = 0.80, as presented in Equation (1):


$$\begin{array}{l} N=\frac{L\left(1-R_{cov}^{2}\right)}{f^{2}} \end{array}$$ (1)

In this equation, *N* is the total sample size, *L* is the noncentrality parameter for the chosen significance level and power, *f*^2^ is Cohen's *f*^2^ effect size, set to *f*^2^ = 0.035, and $R_{cov}^{2}$ is the outcome variance explained by baseline covariates, set to 0.30. G^*^Power 3.1.9.7 gave a minimum total sample of 159, rounded up to *N* = 160; allowing for 15% attrition, the planned sample was *N* = 200, and *N* = 204 participants were recruited, with *n* = 51 randomized to each condition.

Inclusion criteria were graduating undergraduate status, experience with smartphones and professional social networking platforms, and no signed tripartite employment agreement. Students holding a graduate admission offer, majoring in communication, journalism, or an algorithm related field, or having received systematic career planning guidance in the previous 6 months were excluded because they were no longer seeking employment or had already been exposed to content close to the intervention, so the findings apply to job seeking graduates without specialized prior training in media or career guidance.

Randomization was stratified by disciplinary category (engineering, science, and humanities or management), sex, and university and used a computer generated random number sequence from a third party data management platform, and allocation was revealed after the T0 assessment.

### 4.3 Procedure

The career guidance centers of the three universities provided venues and scheduling and cross verified employment documents at T3, six instructors delivered all sessions within the required career planning course, and eight trained graduate research assistants supported data collection and coding.

T0, one week before the intervention, collected self report scales, screen use logs, and DECT performance; T1, on the final day of the intervention, collected the same content; T2, 4 weeks later, assessed persistence; and T3, 6 months after graduation, readministered the self report scales and collected the four employment outcome indicators.

Because T3 took place after participants had left campus, contact details were collected at T0 and reconfirmed at T1 and T2, monthly WeChat check ins were held between T2 and T3, and participants received 30, 50, and 100 yuan at T1, T2, and T3. Of the 12 participants who were not retained, 3 left before T1, 3 before T2, and 6 before T3.

The single blind arrangement concerned the research hypotheses rather than the content of instruction: instructors necessarily knew which module they taught, but neither they nor the participants were informed of the hypotheses, data coders did not deliver the intervention, and analysts did not access group labels before estimating the main effects. These safeguards targeted two research participation effects. The Hawthorne effect refers to changes in behavior or self reports that occur because people know they are being studied, and expectancy effects arise when participants or instructors anticipate that a condition will help and respond accordingly (Boot et al., 2013; McCambridge et al., 2014), mainly through awareness of the hypothesis and motivation to meet perceived expectations (Corneille and Lush, 2023). Such effects could have inflated the T1 and T2 self reports, particularly where outcome content overlapped with training. Because all conditions received 16 instructional hours in the same course, from the same instructors, on the same schedules, and with identically designed materials, no group had reason to expect that its own condition would perform better. Expectations were not measured directly, so differential expectations cannot be ruled out completely (Boot et al., 2013), but the T3 outcomes were collected after participants had dispersed, and three of the four were document verified or externally coded and therefore far less sensitive to expectations than the T1 and T2 self reports.

### 4.4 Measures

Because self reports show whether participants believe they have changed rather than whether their behavior has changed, the study combined self report scales, behavioral data, and employment outcomes, as shown in Figure 2.

**Figure 2:**
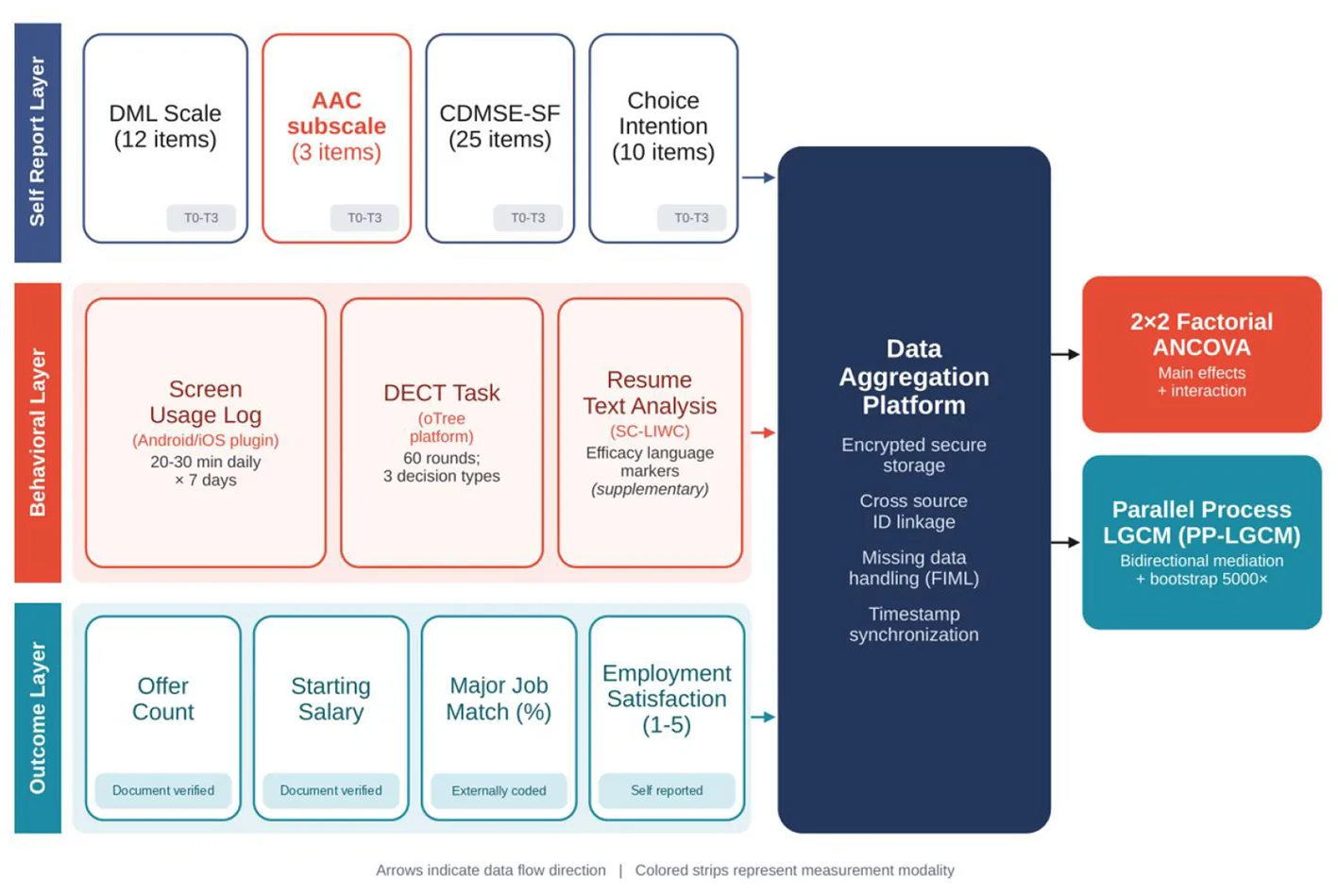
Architecture for multisource multimodal data collection and processing.

All instruments were administered in Simplified Chinese. Items adapted from instruments developed in other cultural contexts, namely the three general digital media literacy subscales and the employment satisfaction scale, were translated following the ITC guidelines for translating and adapting tests (International Test Commission, 2018): two bilingual researchers translated the items independently, a third researcher back translated the reconciled version, discrepancies were resolved by consensus, and six experts in career counseling, communication studies, and educational psychology reviewed the items for cultural relevance, whereas the CDMSE-SF was administered in its existing Chinese version. Items referring to media use named platform types that Chinese graduates use for job search, such as short video and professional networking platforms, rather than specific Western services. All instruments were pilot tested with *n* = 50 students to check item comprehension and internal consistency (Cronbach's α > 0.80 for every instrument); because 50 cases cannot support stable estimation of a multifactor model, these indices were treated as preliminary, and structural validity was evaluated in the formal sample.

#### 4.4.1 Digital media literacy scale

The scale assessed students' capacity to obtain, evaluate, and create media content and to understand how algorithms filter career information. With reference to a six dimensional AI literacy framework (Yuan et al., 2024), the 12 item scale covered information, critical, creation, and algorithmic awareness literacy, with three items each. Items for the information, critical, and creation subscales were adapted from the functional consumption, critical consumption, and functional prosumption factors of the New Media Literacy Scale developed with university students in Turkey (Koc and Barut, 2016), using the translation procedure described above, whereas the AAC subscale was developed for this study. Items were rated from 1 (strongly disagree) to 5 (strongly agree), and subscale scores were the means of their items. In the formal sample, the four factor model fitted well (χ^2^ = 82.1, df = 48, CFI = 0.94, TL I= 0.92, RMSEA = 0.061, SRMR = 0.047), and across the subscales Cronbach's α ranged from 0.81 to 0.87, composite reliability from 0.83 to 0.88, and average variance extracted from 0.62 to 0.71; discriminant validity is reported with the results.

#### 4.4.2 Algorithmic awareness for careers subscale

The AAC subscale was developed following the define, operationalize, and confirm sequence for new constructs (Lambert and Newman, 2023). AAC was defined as students‘ metacognitive capacity to understand how recommendation algorithms filter career information and specified through three facets with one item each, namely perceived algorithmic visibility, critical evaluation of algorithmic outputs, and diversification of algorithmic information, which kept the subscale parallel to the other subdimensions and brief enough for repeated administration. The items were drafted from semi structured interviews with twenty graduating undergraduates and from existing algorithm literacy scales (Dogruel et al., 2022; Gruber and Hargittai, 2023; Voorveld et al., 2024), reviewed by the six experts (scale level content validity index of 0.94; item level values from 0.83 to 1.00), and checked in cognitive interviews with eight graduating undergraduates outside the formal sample. Because the items were written in Chinese from Chinese graduates' descriptions of their own job search platforms, no translation into Chinese was required, and the English versions were produced by back translation for reporting. Like the other subscales, the AAC items were rated from 1 (strongly disagree) to 5 (strongly agree), and their mean formed the subscale score. The final Chinese items and their English translations are listed in Supplementary Table S1. All three items loaded strongly on their factor (0.84, 0.87, and 0.82), composite reliability was 0.88, average variance extracted was 0.71, and Cronbach's α was 0.87, 0.88, 0.87, and 0.86 at T0, T1, T2, and T3, with McDonald's ω of 0.88 at T0.

#### 4.4.3 Career decision making self efficacy scale short form

Career decision making self efficacy was measured with the Chinese version of the 25 item CDMSE-SF (Betz et al., 1996) on a 5 point Likert scale. It covers five domains with five items each, namely self appraisal, occupational information, goal selection, planning, and problem solving, and responses from one (no confidence at all) to five (complete confidence) were summed into a total score from 25 to 125. Because the scale was developed in the United States, its structure in Chinese samples was considered: studies of Chinese students have not consistently reproduced the original five factor structure (Hampton, 2005), and among Chinese graduate students the five domains proved so homogeneous that a single total score was recommended (Jin et al., 2012), so the total score was used. In the formal sample, internal consistency was high (Cronbach's α = 0.96, CR = 0.97), and the single factor model fitted acceptably (CFI = 0.93, TLI = 0.92, RMSEA = 0.058, SRMR = 0.041).

#### 4.4.4 Employment choice intention scale

Employment choice intention was measured with a 10 item instrument developed for this study that covers preferences for location, organizational type, industry, and match. It assesses how clearly and on what information basis students have formed preferences on these four facets, which dominate job search decisions in the Chinese labor market, with three items each for location and organizational type and two each for industry and major job match. The items refer to categories salient to Chinese graduates, such as first tier cities vs. home regions and public institutions, state owned enterprises, and private enterprises; they were written in Chinese, reviewed by the six experts, and rated from one (strongly disagree) to five (strongly agree), and the scale score was the item mean. In the formal sample, Cronbach's α was 0.90, composite reliability 0.91, and average variance extracted 0.51, and the one factor model fitted well (CFI = 0.96, TLI = 0.95, RMSEA = 0.052, SRMR = 0.038); the items are listed in Supplementary Table S2.

#### 4.4.5 Behavioral indicators

A lightweight Android and iOS plug in recorded daily short video and professional networking platform use within a 20 to 30 min window over seven days at T0 and T1, together with content categories and the proportion of active searches. The dynamic employment choice task (DECT), built on oTree (Chen et al., 2016) and illustrated in Figure 3, presented 60 job information cards with algorithmic recommendation features and required students to apply, save, or skip within 30 s, yielding decision consistency, skip rate, and response time. Resumes submitted at T3 were analyzed with the Simplified Chinese version of LIWC (SC-LIWC; Zhao et al., 2016) and a self developed lexicon of career efficacy markers; because the lexicon has not been benchmarked against expert judgment, this frequency served only as a descriptive indicator and entered no hypothesis test.

**Figure 3:**
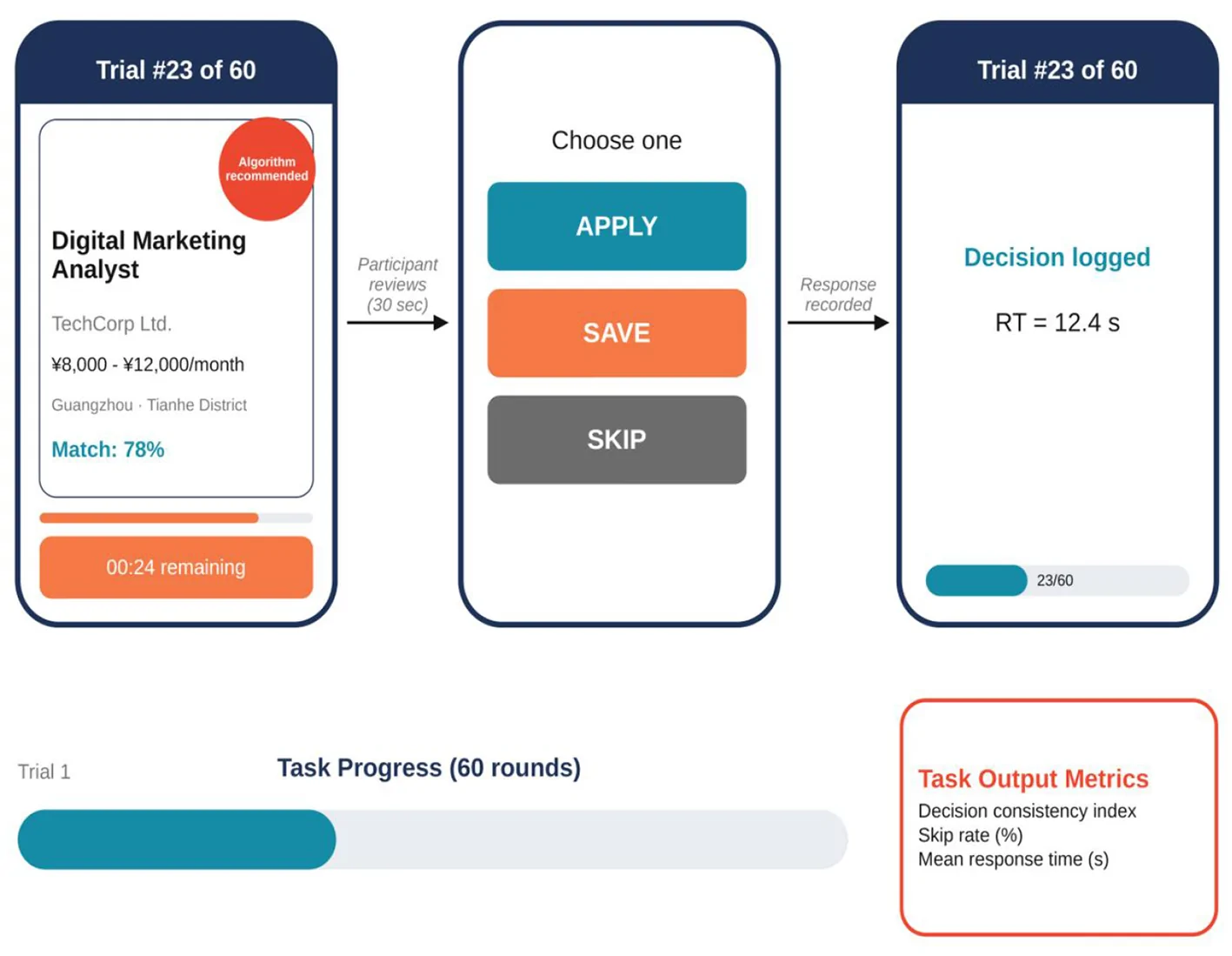
Illustration of the dynamic employment choice task (DECT) scenario.

#### 4.4.6 Employment outcomes

The four employment outcome indicators were collected 6 months after graduation. Participants uploaded their signed employment agreement, offer letter, and first month payslip, which the career guidance centers checked against institutional graduate destination records. Job offers and starting salary were thus document verified, major job match was externally coded from the occupational classification codes of the National Bureau of Statistics, and employment satisfaction was self reported. For major job match, two trained research assistants independently rated on a five level scale (0, 25, 50, 75, and 100) whether the first job fell in the same minor, middle, or major occupational category as the jobs typical of the graduate's major, in a related category, or in an unrelated category; the score was the mean of the two ratings (weighted Cohen's κ = 0.85), and discrepancies of more than one level were resolved by a third coder. Employment satisfaction was measured with the five item overall job satisfaction scale (Judge et al., 1998), translated and adapted as described above and rated from one (strongly disagree) to five (strongly agree); in the T3 sample, Cronbach's α was 0.87 and a one factor model fitted well (CFI = 0.99, TLI = 0.98, RMSEA = 0.058, SRMR = 0.022). Document verification and external coding reduced recall and social desirability bias for the first three indicators, whereas employment satisfaction remains subject to the limitations of self report.

### 4.5 Data analysis

Factorial effects were estimated with a 2 × 2 ANCOVA of T1 scores controlling for T0 scores, and the four conditions were compared on the T3 employment indicators with one way ANOVA and Tukey HSD tests. The factorial model is presented in Equation (2):


$$\begin{array}{l} Y_{ijk}=\mu +\alpha_{i}+\beta_{j}+{\left(\alpha \beta \right)}_{ij}+\gamma X_{ijk}+\epsilon_{ijk} \end{array}$$ (2)

In this equation, *Y*_*ijk*_ is the posttest outcome for participant *k* at MCLT level *i* and CATT level *j*; μ is the grand mean; α_*i*_, β_*j*_, and (αβ)_*ij*_ are the MCLT main effect, the CATT main effect, and their interaction; γ*X*_*ijk*_ is the baseline covariate adjustment; and ϵ_*ijk*_ is the residual.

Dynamic bidirectional mediation was estimated using a parallel process latent growth curve mediation model (PP-LGCM), as presented in Equations (3–7):


$$\begin{array}{lll} \text{AA}{\text{C}}_{t} & = & I_{A}+S_{A}\cdot \lambda_{t}+\zeta_{A,t},\;t\in \left\{T_{0},T_{1},T_{2}\right\} \end{array}$$ (3)

$$\begin{array}{lll} \text{CDMS}{\text{E}}_{t} & = & I_{M}+S_{M}\cdot \lambda_{t}+\zeta_{M,t},\;t\in \left\{T_{0},T_{1},T_{2}\right\} \end{array}$$ (4)

$$\begin{array}{lll} S_{M} & = & a_{0}+a_{1}\cdot I_{A}+a_{2}\cdot S_{A}+\zeta_{S_{M}} \end{array}$$ (5)

$$\begin{array}{lll} \text{AA}{\text{C}}_{T_{3}} & = & d_{0}+d_{1}\cdot I_{A}+d_{2}\cdot S_{A}+d_{3}\cdot S_{M}+\zeta_{A,T_{3}} \end{array}$$ (6)

$$\begin{array}{lll} Y & = & b_{0}+b_{1}\cdot I_{M}+b_{2}\cdot S_{M}+b_{3}\cdot S_{A}+c^{\prime }\cdot G+\zeta_{Y} \end{array}$$ (7)

In this system, AAC_*t*_ and CDMSE_*t*_ are the latent AAC and CDMSE scores at time t, and *I*_*A*_, *S*_*A*_, *I*_*M*_, and *S*_*M*_ are the corresponding intercept and slope factors. The time loading λ_*t*_ reflects the measurement interval in months (λ_*T*_0__ = 0, λ_*T*_1__ = 2, and λ_*T*_2__ = 3), giving unequally spaced linear growth from T0 to T2. AAC_*T*3_ is the AAC score 6 months after graduation, so that *d*_3_ captures the spiral feedback of the CDMSE slope on AAC. *Y* is the composite employment outcome; *a*_1_ and *a*_2_ are the forward paths from the AAC factors to the CDMSE slope; *b*_1_, *b*_2_, and *b*_3_ are the paths from the CDMSE intercept, CDMSE slope, and AAC slope to *Y*; c' is the direct effect of group on *Y*; and ζ denotes residuals. Equation (6) explicitly models the reverse pathway from *S*_*M*_ to AAC_*T*_3__, turning static mediation into a dynamic bidirectional feedback structure. The AAC intercept and slope factors were also regressed on G to estimate the intervention effects on initial status and growth.

The composite indicator *Y* was the unweighted mean of the four T3 indicators after each had been standardized across the T3 sample, as presented in Equation (8):


$$\begin{array}{l} Y_{k}=\frac{1}{Q}\overset{Q}{\underset{q=1}{\sum }}z_{qk} \end{array}$$ (8)

In this equation, *Y*_*k*_ is the composite outcome of participant *k*, $z_{qk}=\frac{x_{qk}-{\bar{x}}_{q}}{s_{q}}$ is the standardized score on indicator *q*, *x*_*qk*_ is the observed value, ${\bar{x}}_{q}$ and *s*_*q*_ are the sample mean and standard deviation of indicator *q*, and *Q* = 4. Equal weights were used because no indicator was theoretically prior.

The significance of indirect effects was tested using a bias corrected bootstrap procedure with 5000 resamples, as presented in Equation (9):


$$\begin{array}{l} \text{C}{\text{I}}_{1-\alpha }\left(\hat{ab}\right)=\left[{\hat{ab}}_{\left(\alpha /2\right)}^{*},\;{\hat{ab}}_{\left(1-\alpha /2\right)}^{*}\right] \end{array}$$ (9)

In this equation, $\hat{ab}$ is the product estimate of the indirect effect, ${\hat{ab}}^{*}$ is its distribution across 5000 bootstrap resamples, and ${\hat{ab}}_{\left(\alpha /2\right)}^{*}$ and ${\hat{ab}}_{\left(1-\alpha /2\right)}^{*}$ are the lower and upper quantiles, whose percentile levels were adjusted for the median bias of the bootstrap distribution; a mediation effect was significant when the interval excluded 0. Analyses were run in Mplus 8.10 (Muthén & Muthén, Los Angeles, CA, USA) and R 4.3.2 (R Core Team, R Foundation for Statistical Computing, Vienna, Austria) with full information maximum likelihood (FIML) for missing data. Because conditions were delivered to session groups, the session level intraclass correlations of the T1 outcomes (0.03 to 0.08; design effects of about 1.5 to 2.2) were handled with a two level model, whereas those of the T3 indicators, collected after participants had dispersed, were below 0.01, so single level analyses were used at T3.

H1 was evaluated with the path from the AAC slope to the composite outcome and with the four condition comparisons at T3; H2 with the indirect and direct effects on the composite outcome; H3 with the path from the AAC slope to the CDMSE slope together with the CATT and MCLT effects on CDMSE; H4 with the path from the CDMSE slope to AAC at T3 [*d*_3_ in Equation (6)]; and H5 with the effect of the intervention on the AAC slope and the indirect effects through the AAC and CDMSE slopes.

## 5 Results

### 5.1 Sample description, scale reliability and validity, and randomization balance tests

Of the 204 undergraduates who completed T0, 192 were retained for analysis after the intervention and three follow up assessments, an attrition rate of 5.9% (12 of 204), below the prespecified 15% threshold. Attrition did not differ across conditions (*A*_1_*B*_1_: 3; *A*_1_*B*_0_: 4; *A*_0_*B*_1_: 2; *A*_0_*B*_0_: 3; χ^2^(3) = 0.71, *p* = 0.871; Fisher-Freeman-Halton exact test, *p* = 0.976). Baseline demographic characteristics are presented in Supplementary Table S3.

Baseline comparability tests showed no significant differences across conditions in age (*M* = 21.6, SD = 0.9), GPA (*M* = 3.24, SD = 0.42), monthly household income, sex, disciplinary distribution, or household registration (all *p* ≥ 0.331). Missing data occurred mainly at T2 and T3, and Little's MCAR test, χ^2^ = 32.7, df = 28, *p* = 0.247, supported the missing completely at random assumption underlying FIML estimation.

As Table 2 shows, all scales met the criteria of CFI > 0.90, RMSEA < 0.08, and SRMR < 0.05, with composite reliability of at least 0.83 and average variance extracted above 0.50. Interfactor correlations between AAC and the other subdimensions (0.31 to 0.48) were below the square roots of the average variance extracted (0.84 for AAC and 0.79 to 0.83 for the others), and the Fisher *z* based 95% confidence interval for the largest correlation, [0.36, 0.58], and the heterotrait monotrait ratios of 0.34, 0.52, and 0.36 remained well below 0.80 (Rönkkö and Cho, 2022), supporting the distinctness of AAC. The factor loadings are shown in Figure 4.

**Table 2:** Internal consistency and confirmatory factor analysis fit indices for each scale.

Scale	Items	Cronbach's α	CR	AVE	CFI	TLI	RMSEA (90% CI)	SRMR
Total digital media literacy	12	0.87	0.93	0.67	0.94	0.92	0.061 (0.037, 0.083)	0.047
Information literacy subdimension	3	0.83	0.85	0.65	NA	NA	NA	NA
Critical literacy subdimension	3	0.86	0.87	0.69	NA	NA	NA	NA
Creation literacy subdimension	3	0.81	0.83	0.62	NA	NA	NA	NA
Algorithmic awareness for careers (AAC)	3	0.87	0.88	0.71	NA	NA	NA	NA
CDMSE-SF	25	0.96	0.97	0.56	0.93	0.92	0.058 (0.049, 0.068)	0.041
Employment choice intention	10	0.90	0.91	0.51	0.96	0.95	0.052 (0.019, 0.079)	0.038

NA, not applicable; the fit of the four subdimensions is that of the four factor model reported for the total scale.

**Figure 4:**
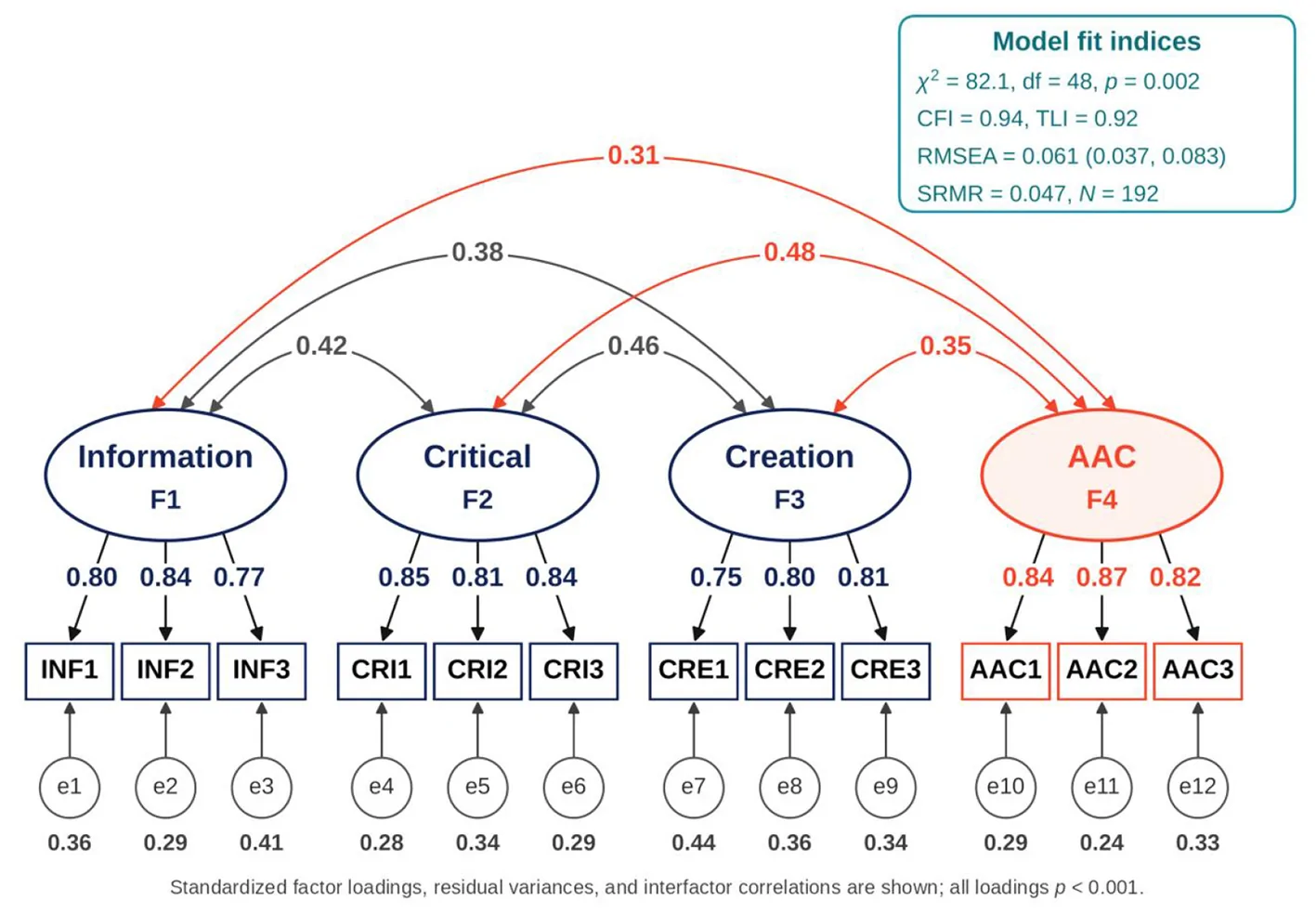
Confirmatory factor analysis path diagram for the four subdimensions of digital media literacy.

### 5.2 Main intervention effects: results of the 2 × 2 factorial ANCOVA

Table 3 presents the 2 × 2 ANCOVA results at T1. CATT had its strongest effect on AAC, $\eta_{p}^{2}$ = 0.128, about 3.5 times that of MCLT, whereas MCLT affected critical literacy more strongly than CATT did, $\eta_{p}^{2}$ = 0.096 vs. 0.019, and information and creation literacy, which neither component targeted, showed smaller effects, supporting the correspondence between intervention content and construct.

**Table 3:** Estimated main and interaction effects from the 2 × 2 factorial ANCOVA at T1.

Outcome variable	MCLT main effect *F*(1,187)	*p*	$\eta_{p}^{2}$	CATT main effect (1,187)	*p*	$\eta_{p}^{2}$	Interaction effect *F*(1,187)	*p*	$\eta_{p}^{2}$
AAC	7.14	0.008	0.037	27.45	< 0.001	0.128	5.28	0.023	0.027
Critical literacy	19.86	< 0.001	0.096	3.65	0.058	0.019	2.28	0.133	0.012
Information literacy	10.75	0.001	0.054	6.42	0.012	0.033	1.98	0.161	0.010
Creation literacy	4.58	0.034	0.024	3.31	0.070	0.017	1.05	0.307	0.006
Total CDMSE	12.63	< 0.001	0.063	17.85	< 0.001	0.087	5.98	0.015	0.031
Employment choice intention	9.14	0.003	0.047	14.15	< 0.001	0.070	4.53	0.035	0.024

For total CDMSE and employment choice intention, both components had significant effects and the interactions indicated positive synergy (*p* = 0.015 and *p* = 0.035), so the combined effect exceeded the sum of the separate effects. The interaction on AAC was significant but negative, consistent with a ceiling effect: the *A*_1_*B*_1_ mean of 4.36 lay only 1.0 standard deviation below the scale maximum, a normal approximation places 23.8% of *A*_1_*B*_1_ and 22.6% of *A*_0_*B*_1_ but 2.1% of *A*_0_*B*_0_ at the maximum, and the increment associated with MCLT fell from 0.72 without CATT to 0.05 with CATT, whereas CDMSE, whose *A*_1_*B*_1_ mean lay 2.09 standard deviations below its maximum, showed positive synergy; content overlap between the modules and the fixed module order may also have contributed. The T1 means and 95% confidence intervals are shown in Figure 5.

**Figure 5:**
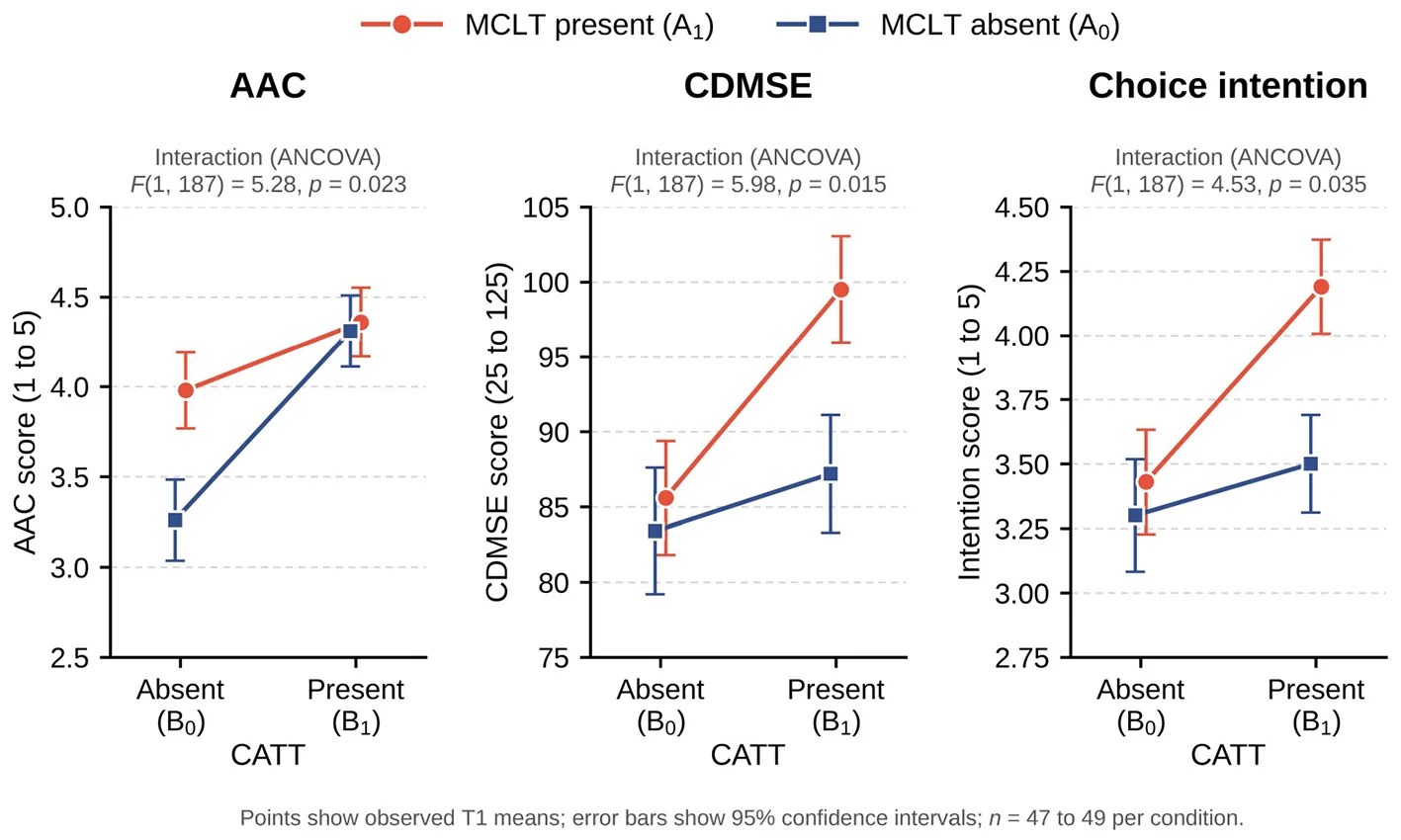
Observed T1 means and 95% confidence intervals for three key outcome variables across the four conditions in the 2 × 2 factorial design.

Relative to *A*_0_*B*_0_, the combined condition reached Hedges' *g* = 1.54 [1.09, 1.99] on AAC, 1.20 [0.77, 1.63] on total CDMSE, and 1.28 [0.85, 1.71] on employment choice intention at T1, whereas CATT alone approached the combined effect only on AAC and single components had small effects on the other two outcomes; condition means, standard deviations, and all effect sizes are reported in Supplementary Table S4. At T2, the combined vs. control *g* attenuated to 1.38 [0.93, 1.83], 1.05 [0.62, 1.48], and 1.18 [0.75, 1.61], a reduction of 8% to 13% from T1, while all three differences remained significant (*p* < 0.01).

### 5.3 Dynamic bidirectional mediation: identification of the spiral feedback pathway with PP-LGCM

The overall fit indices of the parallel process latent growth curve mediation model were χ^2^ = 137.5, df = 87, *p* < 0.001, χ^2^/*df* = 1.58, CFI = 0.958, TLI = 0.945, RMSEA = 0.055 with a 90% CI of [0.037, 0.072], and SRMR = 0.047. All indices met the recommended thresholds. Key path coefficients and 95% bias corrected bootstrap confidence intervals are presented in Table 4.

**Table 4:** Key PP-LGCM path coefficients and 95% bias corrected bootstrap confidence intervals.

Path	Standardized Coefficient β	SE	*z*	*p*	95% CI
Intervention group to AAC intercept *I*_*A*_	0.05	0.072	0.69	0.487	[−0.09, 0.19]
Intervention group to AAC slope *S*_*A*_	0.47	0.074	6.35	< 0.001	[0.32, 0.62]
AAC slope *S*_*A*_ to CDMSE slope *S*_*M*_	0.42	0.083	5.06	< 0.001	[0.26, 0.58]
CDMSE slope *S*_*M*_ to endpoint AAC at T3	0.28	0.077	3.64	< 0.001	[0.13, 0.43]
CDMSE slope *S*_*M*_ to employment outcome	0.36	0.090	4.00	< 0.001	[0.18, 0.54]
AAC slope *S*_*A*_ to employment outcome *Y*	0.22	0.083	2.65	0.008	[0.06, 0.38]
Intervention to employment outcome, direct effect c'	0.13	0.091	1.43	0.153	[−0.05, 0.31]
Indirect effect: AAC slope to CDMSE slope to employment	0.151	0.041	NA	NA	[0.077, 0.238]
Indirect effect: AAC slope to CDMSE slope to endpoint AAC (spiral feedback)	0.118	0.036	NA	NA	[0.055, 0.197]

NA, not applicable; indirect effects were evaluated with bias corrected bootstrap confidence intervals.

The path from the AAC slope to the CDMSE slope was significant, β = 0.42, as was the reverse path from the CDMSE slope to the T3 AAC score, β = 0.28, although the paths among growth factors were not themselves randomized. Both the forward indirect effect on employment outcomes, 0.151, 95% CI [0.077, 0.238], and the reverse indirect pathway to endpoint AAC, 0.118, 95% CI [0.055, 0.197], excluded 0. The direct effect of the intervention on employment outcomes, c' = 0.13, was not significant [95% CI (−0.05, 0.31), *p* = 0.153]. With a standard error of 0.091, the model had about 30% power for a direct effect of this size and 80% power only for effects of about 0.25 or larger, and the 90% confidence interval, [−0.02, 0.28], rules out only direct effects larger than 0.28, so the result is best described as an indirect only pattern in which a small direct effect or additional mediators cannot be excluded (Rohrer et al., 2022). The observed trajectories of AAC and CDMSE are shown in Figure 6.

**Figure 6:**
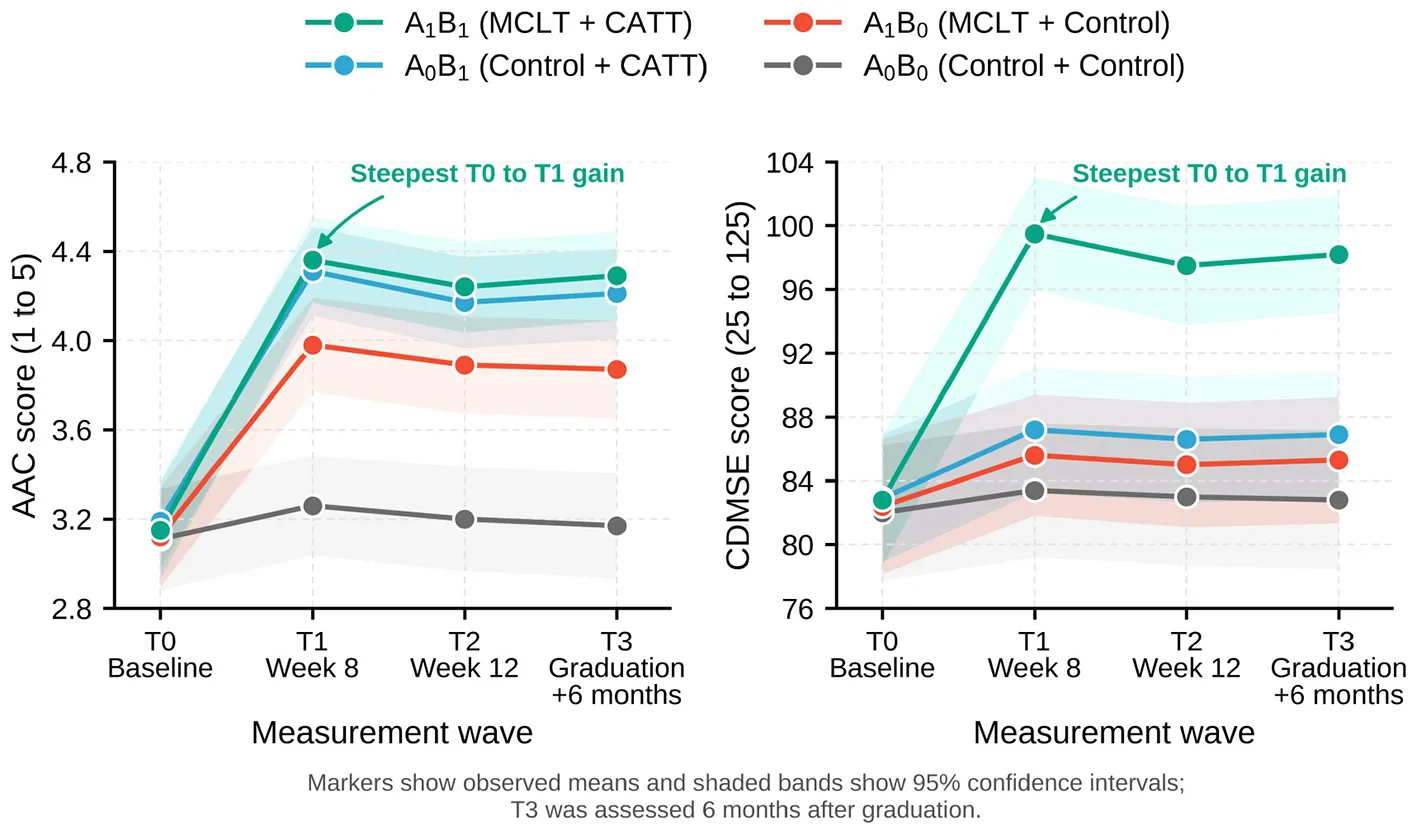
Observed trajectories of AAC and CDMSE across the four conditions.

### 5.4 Intergroup differences in actual employment outcomes and robustness tests

Table 5 compares the four conditions on the employment outcomes 6 months after graduation. The *A*_1_*B*_1_ condition had the highest mean on all four indicators and, compared with *A*_0_*B*_0_, an advantage of 1.44 job offers, 1,137 yuan per month in starting salary, 17.9 percentage points in major job match, and 0.88 points in employment satisfaction [Tukey HSD tests, all *p* < 0.01; Hedges' *g* = 1.19 (0.76, 1.62), 0.81 (0.39, 1.23), 1.14 (0.71, 1.57), and 1.12 (0.69, 1.55), respectively]. Values for *A*_1_*B*_0_ and *A*_0_*B*_1_ fell between these conditions and did not differ from each other, *p* > 0.10, and each single component produced less than 60% of the advantage of the combined condition, which parallels the synergy observed for CDMSE at T1 (Figure 7).

**Table 5:** Comparisons of employment outcomes among the four conditions at six months after graduation.

Outcome indicator	*A*_1_*B*_1_ (*n* = 48)	*A*_1_*B*_0_ (*n* = 47)	*A*_0_*B*_1_ (*n* = 49)	*A*_0_*B*_0_ (*n* = 48)	*F* (3,188)	*p*	$\eta_{p}^{2}$
Number of job offers received^*a*^	3.52 (1.25)	2.58 (1.32)	2.83 (1.28)	2.08 (1.16)	10.96	< 0.001	0.149
Starting salary (yuan/month)^*a*^	7,415 (1,310)	6,653 (1,442)	6,835 (1,370)	6,278 (1,492)	5.46	0.001	0.080
Major job match (%)^*b*^	75.1 (14.5)	62.7 (15.8)	67.0 (14.7)	57.2 (16.8)	11.43	< 0.001	0.154
Employment satisfaction (1 to 5)^*c*^	3.98 (0.73)	3.42 (0.80)	3.55 (0.76)	3.10 (0.83)	10.46	< 0.001	0.143

Values in the table are means with standard deviations in parentheses.

^*a*^Document verified indicator.

^*b*^Externally coded indicator.

^*c*^Self reported indicator.

**Figure 7:**
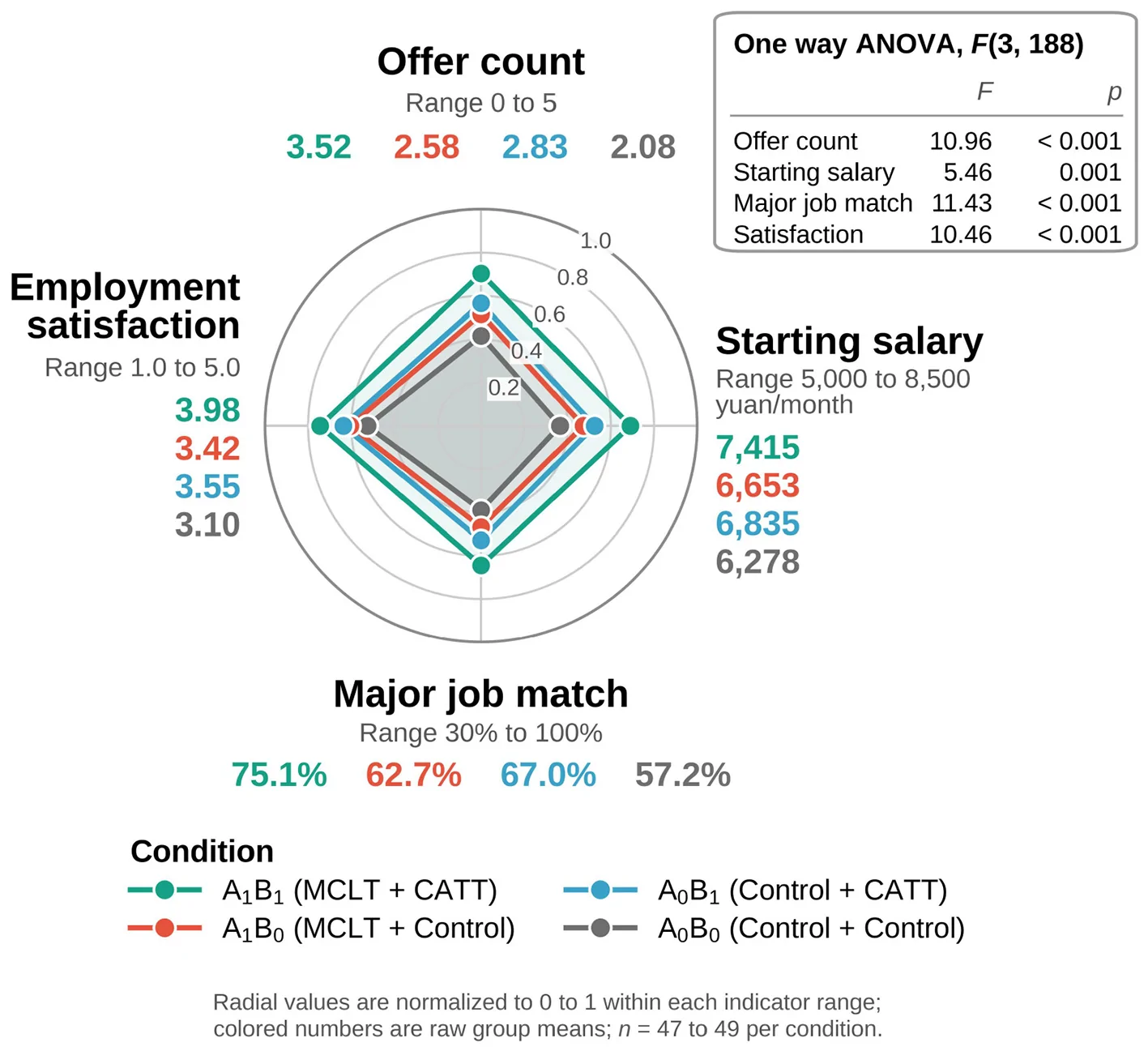
Radar chart comparing employment outcomes across the four conditions.

Sensitivity analyses, comprising exclusion of extreme values, multiple imputation, Bonferroni correction, propensity score matching, and stratification by sex and discipline, are reported in Supplementary Table S5. The key path coefficients and employment outcome differences remained significant and in the same direction, with a slightly larger effect in the humanities and management subsample than in the engineering subsample.

The ancillary behavioral data converged with these results. At T1, *A*_1_*B*_1_ participants reduced daily short video use by 16.3% and increased active career related searches by 42.7%, compared with 2.4% and 5.1% in *A*_0_*B*_0_, and on the DECT they reached 80.5% decision consistency, a 14.1% skip rate, and a mean response time of 10.7 s, compared with 63.8%, 28.6%, and 14.5 s in *A*_0_*B*_0_. Because the plug in captured only within window use and the DECT used constructed job cards, these indicators serve only as convergent evidence.

### 5.5 Summary of hypothesis tests

Table 6 summarizes the evidence for the five hypotheses. H1 and H4 were supported by the forward and reverse growth paths, and H5 was supported because the intervention raised the AAC slope [β = 0.47, 95% CI (0.32, 0.62)] and this growth reached employment outcomes through the reciprocal AAC and CDMSE pathways. H2 was supported in an indirect only form, and H3 received partial support because the PP-LGCM modeled AAC, but not the other three subdimensions, in parallel with CDMSE.

**Table 6:** Summary of research hypothesis tests.

Hypothesis	Summary	Key evidence	Result
H1	Digital media literacy positively predicts the quality of employment choices	AAC slope to *Y* β = 0.22, 95% CI [0.06, 0.38]; *A*_1_*B*_1_ higher than *A*_0_*B*_0_ on all four employment indicators; indirect effect β = 0.151, 95% CI [0.077, 0.238]	Supported
H2	CDMSE partially mediates the relationship between digital media literacy and the quality of employment choices	Direct effect c' = 0.13, 95% CI [−0.05, 0.31], n.s.; indirect pathway significant	Supported; indirect only pattern, a small direct effect cannot be excluded
H3	The independent effect of AAC on CDMSE exceeds those of the other subdimensions	AAC slope to CDMSE slope β = 0.42, 95% CI [0.26, 0.58]; CATT to CDMSE $\eta_{p}^{2}$ = 0.087 vs. MCLT to CDMSE 0.063; CATT to AAC $\eta_{p}^{2}$ = 0.128; MCLT to critical literacy $\eta_{p}^{2}$ = 0.096; discriminant validity: *r* = 0.31 to 0.48, all below the square roots of AVE	Partially supported; comparison with the other three subdimensions not tested directly
H4	Growth in CDMSE positively predicts subsequent AAC, forming spiral feedback	Reverse pathway β = 0.28, 95% CI [0.13, 0.43]	Supported
H5	Intervention effects are transmitted through the spiral mechanism to actual employment outcomes	Intervention to AAC slope β = 0.47, 95% CI [0.32, 0.62]; *A*_1_*B*_1_ vs. *A*_0_*B*_0_: all four employment outcome indicators had *p* < 0.01	Supported

## 6 Discussion

### 6.1 Findings consistent with previous research

Several findings agree with previous research and extend it, as summarized in Table 7. CDMSE carried the training effect from AAC growth to employment outcomes, in line with studies in which CDMSE transmitted the effects of social support, psychological capital, and career adaptability to career outcomes (Kvasková et al., 2023; Wang et al., 2024b; Xiong et al., 2025; Zhou et al., 2024), and digital literacy was again linked to better employment related outcomes among Chinese university students (Chen et al., 2025; Gao et al., 2025). The advance lies in extending this chain from self reported intentions to verified and externally coded employment outcomes, which reduces, although it does not eliminate, the reliance of SCCT research on self report.

**Table 7:** Comparison of key findings with previous research.

Finding of this study	Comparable evidence in the literature	Comparison	Possible explanation
AAC growth predicted CDMSE growth, and CDMSE carried the training effect to employment outcomes	CDMSE mediates the effects of support, psychological capital, and adaptability on career outcomes (Kvasková et al., 2023; Wang et al., 2024b; Xiong et al., 2025; Zhou et al., 2024)	Consistent	Algorithm training acts as a domain specific learning experience that SCCT places before efficacy
CATT mainly raised AAC, and MCLT mainly raised critical literacy	Training raises the targeted competence (Huang et al., 2026; Lu et al., 2024; Markus et al., 2025)	Consistent	Module content aligned with subscale content
Effects declined by 8% to 13% from T1 to T2	Career intervention gains are not always maintained (Wang et al., 2024a)	Consistent	Gains attenuate without continued practice
CDMSE growth predicted later AAC (β = 0.28)	CDMSE is modeled as a unidirectional mediator (Agoes Salim et al., 2023; Cheung, 2024; Karacan Ozdemir and Buyukcolpan, 2024)	Differs	Growth modeling after randomized induction captures the loop, and recommendation feeds respond to active search
Only an indirect effect was detected	Cross sectional models usually retain a significant direct path (Wang et al., 2024b; Zhou et al., 2024)	Differs	Mostly verified outcomes share little method variance with self reports, and power for small direct effects was limited (Rohrer et al., 2022)
T1 effects exceeded one standard deviation	Typical educational and media literacy effects are small to moderate (Huang et al., 2026; Kraft, 2023; Lu et al., 2024)	Larger	Intensive program, measures aligned with training (Wolf and Harbatkin, 2023), and a most intensive versus control contrast

The component specific effects also agree with intervention research: CATT mainly raised AAC and MCLT mainly raised critical literacy, matching the target specificity of media literacy and algorithm awareness training (Huang et al., 2026; Lu et al., 2024; Markus et al., 2025), and the discriminant validity of AAC agrees with research that treats algorithm awareness as a construct with its own indicators (Dogruel et al., 2022; Gruber and Hargittai, 2023). The 8% to 13% decline in effect sizes from T1 to T2 likewise matches review evidence that career intervention gains are not always maintained without continued practice (Wang et al., 2024a).

### 6.2 Findings that differ from previous research

The clearest departure from previous work is the reverse path from CDMSE growth to later AAC. Applied SCCT studies have almost always modeled CDMSE as a unidirectional mediator (Agoes Salim et al., 2023; Cheung, 2024; Karacan Ozdemir and Buyukcolpan, 2024), leaving the feedback assumption of the theory untested (Wang et al., 2022; Yao and McWha-Hermann, 2025). Theoretically, randomized induction combined with growth modeling makes this loop empirically visible. Situationally, recommendation systems respond to user behavior, so students whose efficacy grew were more likely to search actively, compare sources, and adjust settings, and thereby reshaped the algorithmic information they subsequently saw (Erickson, 2024; Oeldorf-Hirsch and Neubaum, 2023). Because the paths among growth factors were not randomized, this remains a plausible interpretation rather than a proof of mechanism.

Two further results differ from typical findings. Cross sectional studies of CDMSE usually recover a significant direct effect alongside the indirect pathway (Wang et al., 2024b; Zhou et al., 2024), whereas only an indirect effect was found here, because the time separated composite rests mainly on verified indicators that share little method variance with the self reported predictors and because power to detect a small direct effect was limited (Rohrer et al., 2022). The combined vs. control effects at T1 (Hedges' *g* from 1.20 to 1.54) also exceeded the small effects typical of educational trials and the average of about half a standard deviation in media literacy meta analyses (Huang et al., 2026; Kraft, 2023; Lu et al., 2024); multiple sessions across eight weeks, measures aligned with training content (Wolf and Harbatkin, 2023), and the contrast between the most intensive condition and the control plausibly enlarged these estimates, and the T3 effects on verified indicators were accordingly smaller (*g* from 0.81 to 1.19). The negative interaction on AAC likewise departs from the additive or synergistic pattern expected in factorial trials (White et al., 2024) and is most plausibly explained by a ceiling effect.

### 6.3 Theoretical and practical implications

Theoretically, the findings place algorithmic awareness inside the social cognitive account of career choice. Digital media literacy contains an algorithm specific component that can be measured separately and changed by instruction, and whereas algorithm literacy interventions have rarely been evaluated in formal education (Gagrčin et al., 2026) and have targeted other domains such as intelligent voice assistants (Markus et al., 2025), the present study specifies the content, function, and decision domain of algorithmic awareness in career development and shows that media and career interventions work as sets of components with distinct mechanisms whose effects reach verified employment outcomes.

For practice, an eight hour algorithm transparency module can be embedded in existing career planning courses without extending teaching time, and combining it with critical media literacy training produced the largest gains in CDMSE and employment choice intention. The three item AAC subscale can screen students with low awareness of algorithmic filtering at course entry, and platforms could explain why a position is recommended. Because these implications rest on a single experiment in one region, the 18.1% higher starting salary and the 17.9 percentage point increase in major job match warrant replication before adoption at scale or use in employment policy.

## 7 Conclusion

This randomized factorial study examined how digital media literacy, and its algorithmic component in particular, shapes employment choices through career decision making self efficacy. Four conclusions follow.

(1) Algorithmic Awareness for Careers is a distinguishable subdimension of digital media literacy that responds specifically to algorithm transparency training, whereas critical media literacy training mainly strengthens critical literacy.

(2) AAC and CDMSE develop reciprocally: growth in AAC raises CDMSE, and growth in CDMSE in turn heightens later awareness of algorithmic filtering, which empirically operationalizes the triadic reciprocal feedback of social cognitive career theory.

(3) CDMSE is the principal identified pathway through which the training reaches employment outcomes, since the indirect effect was significant and no direct effect was detected; because power to detect small direct effects was limited, a small residual direct effect cannot be excluded.

(4) Combining the two training components is associated with more offers, an 18.1% higher starting salary, closer major job match, and greater employment satisfaction 6 months after graduation.

These conclusions rest on graduating undergraduates from three universities in southern China without prior media or career guidance training, on T1 and T2 self reports collected shortly after training, on a fixed module order, and on a 6 month follow up; replication with broader samples, independent outcome measures, counterbalanced module order, and follow up of one to three years is needed before the findings inform employment policy.

## Data Availability

The original contributions presented in the study are included in the article/Supplementary material, further inquiries can be directed to the corresponding author.
