# Supplementary material for: An experimental study on the mediating roles of career decision-making self-efficacy between digital media literacy and employment choices of university students
Source: Front Psychol. 2026 Oct 22;17:1963363. doi: 10.3389/fpsyg.2026.1963363 (PMC13736706; doi:10.3389/fpsyg.2026.1963363)
Supplement: Supplementary file 1 [file Supplementary_File_1.docx]

Supplementary Table S1. Items of the Algorithmic Awareness for Careers (AAC) Subscale

| Item | Facet | Chinese Wording | English Translation |
| --- | --- | --- | --- |
| AAC1 | Perceived algorithmic visibility | 我能识别短视频或职业社交平台上关于职业与就业的内容是否是根据我的浏览记录或账户信息被算法推荐的。 | I can recognize whether career and employment content on short video or professional networking platforms has been algorithmically recommended based on my browsing history or account information. |
| AAC2 | Critical evaluation of algorithmic outputs | 当看到平台算法推荐的就业或行业信息时，我会主动判断这些信息可能是平台筛选的结果，未必反映完整的职业机会。 | When I see employment or industry information recommended by a platform algorithm, I actively consider that this information may reflect platform filtering and may not represent the complete set of career opportunities. |
| AAC3 | Diversification of algorithmic information | 我会主动调整平台推荐设置或跨多个渠道检索职业信息，以获取算法通常不会推荐给我的就业内容。 | I actively adjust platform recommendation settings or search across multiple channels to obtain career information that the algorithm would not typically recommend to me. |

Note: Each item was rated on a five point scale from 1 (strongly disagree) to 5 (strongly agree). The AAC score is the mean of the three item ratings, and higher scores indicate greater algorithmic awareness for careers. AAC is one of the four subdimensions of the 12 item digital media literacy scale described in the Measures section of the main text, and its reliability and factor loadings are reported in Table 2 and Figure 4.

Supplementary Table S2. Items of the Employment Choice Intention Scale

| Item | Facet | Chinese Wording | English Translation |
| --- | --- | --- | --- |
| ECI1 | Location | 我清楚自己希望在哪个城市或地区就业。 | I know clearly in which city or region I want to work. |
| ECI2 | Location | 我已经权衡过在一线城市就业与回生源地就业的利弊。 | I have weighed the advantages and disadvantages of working in a first tier city against returning to my home region. |
| ECI3 | Location | 我对就业地点的偏好建立在充分的信息基础上。 | My preference for a work location is based on sufficient information. |
| ECI4 | Organizational type | 我清楚自己更倾向于机关事业单位、国有企业还是民营企业。 | I know clearly whether I prefer public institutions, state owned enterprises, or private enterprises. |
| ECI5 | Organizational type | 我了解不同类型用人单位在发展机会和稳定性方面的差异。 | I understand how different types of employers differ in development opportunities and stability. |
| ECI6 | Organizational type | 我对用人单位类型的选择与自己的职业目标一致。 | My choice of employer type is consistent with my career goals. |
| ECI7 | Industry | 我已经确定了一至两个目标行业。 | I have identified one or two target industries. |
| ECI8 | Industry | 我能够较准确地判断目标行业的发展前景。 | I can judge the prospects of my target industries fairly accurately. |
| ECI9 | Major job match | 我清楚哪些岗位与自己的专业相匹配。 | I know clearly which positions match my major. |
| ECI10 | Major job match | 我能够判断一个岗位对专业知识的要求是否与我所学相符。 | I can judge whether the knowledge a position requires matches what I have studied. |

Note: Items were rated from 1 (strongly disagree) to 5 (strongly agree); the scale score is the mean of the ten items, and higher scores indicate clearer and better informed employment preferences.

Supplementary Table S3. Baseline Demographic Characteristics of the Sample ($N$=192)

| Variable | Category | Frequency | Percentage |
| --- | --- | --- | --- |
| Sex | Male | 94 | 49.0% |
|  | Female | 98 | 51.0% |
| Disciplinary Category | Engineering | 68 | 35.4% |
|  | Science | 47 | 24.5% |
|  | Humanities and Management | 77 | 40.1% |
| Household Registration | Urban | 106 | 55.2% |
|  | Rural | 86 | 44.8% |
| Only Child | Yes | 79 | 41.1% |
|  | No | 113 | 58.9% |
| GPA Group | ≥3.5 | 58 | 30.2% |
|  | 3.0 to 3.5 | 96 | 50.0% |
|  | <3.0 | 38 | 19.8% |
| Monthly Household Income (yuan) | ≤6000 | 43 | 22.4% |
|  | 6000 to 15000 | 108 | 56.3% |
|  | >15000 | 41 | 21.4% |

Supplementary Table S4. Observed Means, Standard Deviations, and Hedges' $g$ Relative to the Control Condition at T1 and T2

| Outcome | Wave and Statistic | $A_{1}B_{1}$ | $A_{1}B_{0}$ | $A_{0}B_{1}$ | $A_{0}B_{0}$ |
| --- | --- | --- | --- | --- | --- |
| AAC | T1 M (SD) | 4.36 (0.66) | 3.98 (0.72) | 4.31 (0.69) | 3.26 (0.77) |
|  | T1 $g$ [95% CI] | 1.54 [1.09, 1.99] | 0.97 [0.55, 1.39] | 1.44 [1.00, 1.88] | Reference |
|  | T2 M (SD) | 4.24 (0.70) | 3.89 (0.74) | 4.17 (0.71) | 3.20 (0.80) |
|  | T2 $g$ [95% CI] | 1.38 [0.93, 1.83] | 0.90 [0.47, 1.32] | 1.28 [0.85, 1.72] | Reference |
| Total CDMSE | T1 M (SD) | 99.5 (12.2) | 85.6 (12.9) | 87.2 (13.6) | 83.4 (14.5) |
|  | T1 $g$ [95% CI] | 1.20 [0.77, 1.63] | 0.16 [-0.24, 0.56] | 0.27 [-0.13, 0.67] | Reference |
|  | T2 M (SD) | 97.5 (12.8) | 85.0 (13.3) | 86.6 (13.8) | 83.0 (14.8) |
|  | T2 $g$ [95% CI] | 1.05 [0.62, 1.48] | 0.14 [-0.26, 0.54] | 0.25 [-0.15, 0.65] | Reference |
| Employment Choice Intention | T1 M (SD) | 4.19 (0.63) | 3.43 (0.69) | 3.50 (0.66) | 3.30 (0.75) |
|  | T1 $g$ [95% CI] | 1.28 [0.85, 1.71] | 0.18 [-0.22, 0.58] | 0.28 [-0.12, 0.68] | Reference |
|  | T2 M (SD) | 4.12 (0.65) | 3.40 (0.70) | 3.46 (0.67) | 3.29 (0.75) |
|  | T2 $g$ [95% CI] | 1.18 [0.75, 1.61] | 0.15 [-0.25, 0.55] | 0.24 [-0.16, 0.64] | Reference |

Note: $g$ = Hedges' $g$ relative to $A_{0}B_{0}$ with 95% confidence intervals in brackets; $n$ = 48, 47, 49, and 48 in $A_{1}B_{1}$, $A_{1}B_{0}$, $A_{0}B_{1}$, and $A_{0}B_{0}$; T1 was assessed on the final day of the intervention and T2 4 weeks later.

Supplementary Table S5. Sensitivity Analysis Results From the Robustness Tests

| Testing Scheme | Analytical Method | AAC Slope to CDMSE Slope $\beta$ [95% CI] | Employment Outcome Difference $F$ | Robustness of Conclusion |
| --- | --- | --- | --- | --- |
| Full Sample | PP-LGCM | 0.42 [0.26, 0.58] | 10.96 | Reference |
| Exclusion of Extreme Values (±3SD) | PP-LGCM | 0.39 [0.23, 0.55] | 10.58 | Robust |
| Multiple Imputation (20 times) | MICE + PP-LGCM | 0.41 [0.24, 0.57] | 11.17 | Robust |
| Bonferroni Correction | ANCOVA | NA | $p$<0.001 | Robust |
| Propensity Score Matching | PSM + ANCOVA | 0.38 [0.21, 0.55] | 10.31 | Robust |
| Subsamples by Sex | Stratified PP-LGCM | Male 0.40 / Female 0.44 | Male 5.09 / Female 5.95 | Robust |
| Subsamples by Discipline | Stratified PP-LGCM | Engineering 0.39 / Science 0.43 / Humanities and Management 0.46 | Engineering 3.86 / Science 3.74 / Humanities and Management 5.84 | Robust |

Note: NA = not applicable..
